# Nutrition and Micronutrient Interactions in Autoimmune Thyroid Disorders: Implications for Cardiovascular Health

**DOI:** 10.3390/pathophysiology32030037

**Published:** 2025-08-01

**Authors:** Michał Mazur, Magdalena Szymańska, Agnieszka Malik, Wojciech Szlasa, Joanna Popiołek-Kalisz

**Affiliations:** 1 Lifestyle Medicine Students’ Club, Medical University of Lublin, ul. Chodzki 7, 20-093 Lublin, Poland; 2 Clinical Dietetics Unit, Medical University of Lublin, 20-093 Lublin, Poland; magdalena.szymanska@umlub.pl (M.S.); joanna.popiolek-kalisz@umlub.pl (J.P.-K.); 3 Department of Biotechnology, Microbiology and Human Nutrition, Faculty of Food Sciences and Biotechnology, University of Life Sciences in Lublin, 20-704 Lublin, Poland; agnieszka.malik@up.lublin.pl; 4 Faculty of Medicine, Wroclaw Medical University, Mikulicza-Radeckiego 5, 50-345 Wroclaw, Poland; 5 Department of Cardiology, Cardinal Wyszynski Hospital in Lublin, 20-718 Lublin, Poland

**Keywords:** thyroid hormones, cardiovascular system, Mediterranean diet, dietary intervention, micronutrients, Hashimoto’s

## Abstract

Thyroid hormones play a crucial role in regulating metabolism and cardiovascular function, with even mild dysfunction—such as subclinical hypothyroidism—negatively impacting heart health. While previous studies have confirmed the effects of iodine, selenium, and vitamin D on thyroid regulation and inflammation, the combined role of these nutrients in reducing cardiovascular disease (CVD) risk in autoimmune thyroid disorders remains insufficiently understood. This review explores the influence of specific micronutrients—including selenium, iodine, and zinc—and dietary patterns, particularly the Mediterranean diet, on the pathophysiology of hypothyroidism and Hashimoto’s thyroiditis. We introduce a novel framework that integrates emerging data on sex-specific micronutrient interactions and nutritional immunomodulation. Unlike the existing literature, this review introduces original hypotheses related to sex-specific nutritional immunomodulation and proposes a novel framework for micronutrient-driven dietary intervention in Hashimoto’s thyroiditis.

## 1. Introduction

Thyroid hormones are essential regulators of key physiological processes, including the cardiovascular system, by maintaining homeostasis through the regulation of heart rate, blood pressure, and lipid profiles. Their actions also encompass metabolic processes, thermoregulation, as well as growth and development. Both deficiency and excess of thyroid hormones could lead to severe health disorders, including cardiovascular diseases (CVD) [1]. In recent decades, increasing evidence highlighted the significant impact of thyroid dysfunction on the cardiovascular system. Both subclinical and overt hypothyroidism (OH) induce changes in the electrocardiogram and cause disturbances in the function of the atria and ventricles. Patients with hypothyroidism exhibited, among other things, greater QT interval dispersion, reduced heart rate variability, and abnormalities during the diastolic phase [2]. In more severe cases, life-threatening conditions such as AV block may even occur [3]. In the former, hormones interact with nuclear receptors, while in the latter, they may act independently of these receptors, directly affecting the cell membranes of cardiomyocytes and blood vessels [4]. This influence was further modulated by factors such as abnormal lipid levels, chronic low-grade inflammation, increased oxidative stress, and insulin resistance, all of which contribute to the strong association between hypothyroidism and CVD risk [5]. Hypothyroidism is classified as primary or secondary, with primary causes including iodine deficiency, autoimmune diseases (e.g., Hashimoto’s thyroiditis), enzymatic damage, radiotherapy, thyroid surgery, certain medications, and chronic or infiltrative conditions, while secondary hypothyroidism results from pituitary or hypothalamic disorders. Diagnosis is typically based on Thyroid-Stimulating Hormone (TSH) levels: subclinical hypothyroidism is characterized by elevated TSH with normal triiodothyronine (T3) and thyroxine (T4), and overt cases showed elevated TSH levels with low T3 and T4. The condition affected prolactin levels and ovulation, making its recognition and treatment especially important during pregnancy [6,7,8]. This review aims to examine how specific nutrients and trace elements—particularly selenium, iodine, zinc, iron, and vitamins D and E—influence both thyroid and cardiovascular function in individuals with hypothyroidism and Hashimoto’s disease. While the existing literature often addresses general dietary recommendations, few reviews integrate molecular mechanisms, population-level data, and interventional evidence linking nutrition with thyroid autoimmunity and cardiovascular risk. To address this gap, we provide new clinical insight into how targeted nutritional strategies—such as precision micronutrient supplementation and adherence to the Mediterranean diet—may prevent or reduce cardiovascular complications in patients with autoimmune thyroid disorders. We also highlight emerging evidence supporting the development of testable hypotheses and propose a framework for personalized dietary interventions. By synthesizing recent findings from endocrinology, nutrition science, and cardiometabolic research, the aim is to integrate nutritional and clinical evidence to guide future research. While previous reviews have addressed micronutrient roles in thyroid dysfunction or cardiovascular risk independently, this review offers a novel integrative approach by linking nutrient status, sex-specific effects, and autoimmune mechanisms into a unified framework. We propose a sex-differentiated, micronutrient-focused model that has not been described in the existing literature.

### 1.1. Research Hypotheses

Considering the developing links among thyroid issues, cardiovascular risks, and nutritional influences, we suggest the subsequent research hypotheses to direct upcoming investigations:

**Hypothesis 1:**  *Targeted dietary interventions rich in selenium, zinc, iodine, and vitamin D can reduce cardiovascular risk in individuals with subclinical hypothyroidism or Hashimoto’s thyroiditis.*

Selenium has been shown to reduce TPOAb and TgAb levels (see Section 6.4 for clinical evidence).

**Hypothesis 2:**  *Adherence to a Mediterranean dietary pattern appears to influence thyroid autoimmunity—indicated by lower levels of TPOAbs and TgAbs—and may decrease systemic inflammation, subsequently improving cardiovascular outcomes.*

See Section 5.4 for a detailed discussion.

**Hypothesis 3:**  *Tailored micronutrient supplementation targeting specific deficiencies results in more significant enhancements in thyroid function and cardiovascular indicators than generic supplementation methods.*

Current studies often apply fixed-dose supplementation (e.g., 200 μg selenium/day) without accounting for the baseline status. Naliwajko et al. [9] found varying micronutrient deficiencies in Polish women with Hashimoto’s disease, suggesting the need for tailored approaches. Our hypothesis calls for precision nutrition models that stratify patients based on biochemical profiles and sex-specific responses—generating new paradigms for thyroid-CVD comanagement [9].

**Hypothesis 4:**  *The cardiometabolic reaction to dietary changes in thyroid function differs by sex, exhibiting varied effects in male and female individuals with autoimmune thyroid diseases.*

Studies by Ghasemi et al. [10] and Vinceti et al. [11] showed sex differences in trace element metabolism and cardiovascular outcomes. Selenium has shown both protective and hypertensive effects in women, while zinc appears protective in females but risk-increasing in males with metabolic syndrome (MetS). This hypothesis introduces a novel sex-differentiated perspective into dietary intervention research in thyroid disease—a currently underexplored area [10,11].

These hypotheses emphasize the promise of dietary approaches as additional methods for addressing thyroid-associated cardiovascular risk, and they necessitate focused clinical trials and mechanistic research to confirm the noted connections.

### 1.2. Molecular Basis of Thyroid Hormone Influence on Cardiovascular Homeostasis

Thyroid hormones, especially T3, exert a profound influence on cardiovascular physiology through both genomic and non-genomic mechanisms [12]. Genomic effects are mediated by nuclear thyroid hormone receptors (THRs), primarily TRα1 in the heart and vasculature. Upon T3 binding, these receptors regulate the transcription of genes involved in cardiomyocyte contractility, hypertrophy, and metabolism. For example, T3 upregulates expression of the α-myosin heavy chain (α-MHC), sarcoplasmic reticulum Ca^2^⁺ ATPase (SERCA2), and Na⁺/K⁺-ATPase, enhancing myocardial efficiency and diastolic function [13,14,15]. Non-genomic actions occur independently of transcriptional regulation and involve interaction with plasma membrane receptors such as integrin αvβ3 [16]. These signaling cascades play a key role in non-genomic regulation of cardiovascular function. MAPK pathways regulate cardiomyocyte proliferation, stress responses, and inflammatory gene expression, while the PI3K/Akt pathway contributes to endothelial nitric oxide production, cell survival, and the inhibition of apoptosis [17,18]. Zinc plays a structural and regulatory role in numerous transcription factors and antioxidant enzymes, while iron deficiency impairs thyroid hormone synthesis and is associated with worsening cardiovascular outcomes [19,20].

**Figure 1:**
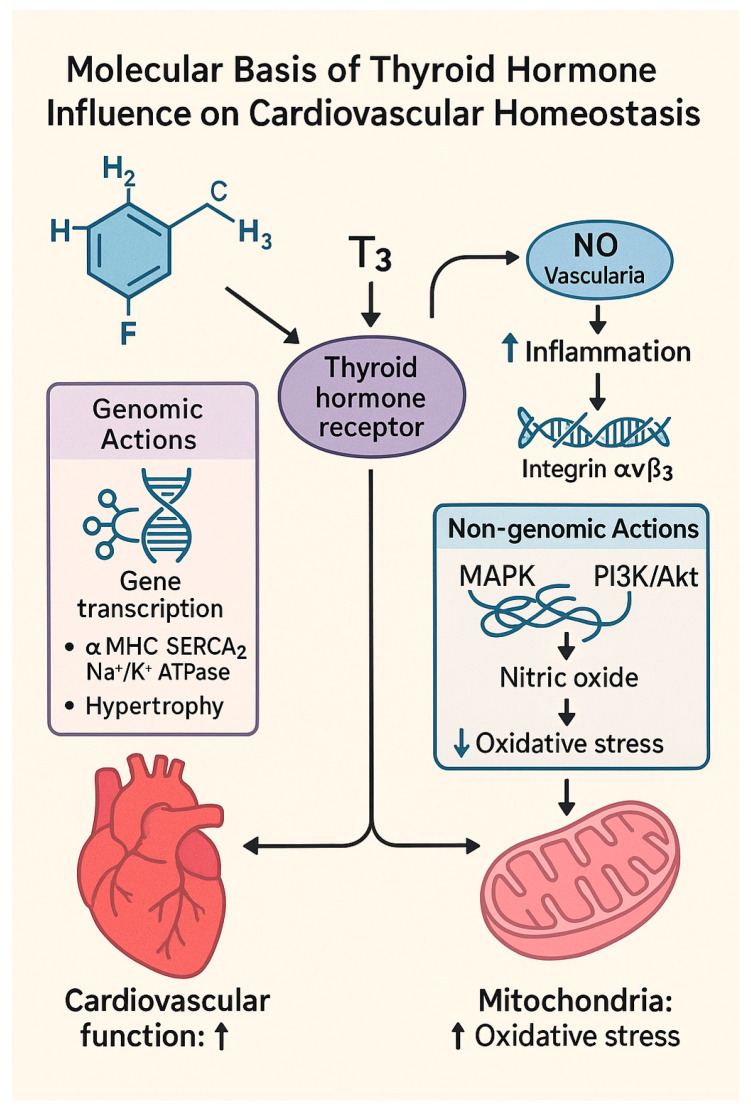
Molecular basis of thyroid hormone (T3) influence on cardiovascular homeostasis. T3 binds to the thyroid hormone receptor and triggers both genomic and non-genomic actions. Left pathway (genomic): T3–receptor complex activates gene transcription, upregulating αMHC, SERCA_2_, and Na⁺/K⁺ ATPase, which leads to cardiac hypertrophy and enhanced cardiovascular function. Right pathway (non-genomic): T3 modulates endothelial nitric oxide (NO) production via PI3K/Akt and MAPK signaling, reducing systemic inflammation and oxidative stress, which in turn affects mitochondrial function. Arrows indicate the direction of molecular signaling and physiological consequences of T3 action. Original graphic created by the authors using elements and icons licensed under Canva subscription. No third-party copyrighted content was used.

Importantly, these pathways are activated rapidly and independently of nuclear gene transcription. As illustrated in Figure 1, T3 influences cardiovascular function via both genomic and non-genomic pathways integrate with genomic actions to maintain vascular tone, limit oxidative stress, and improve mitochondrial function.

## 2. Materials and Methods

**Figure 2:**
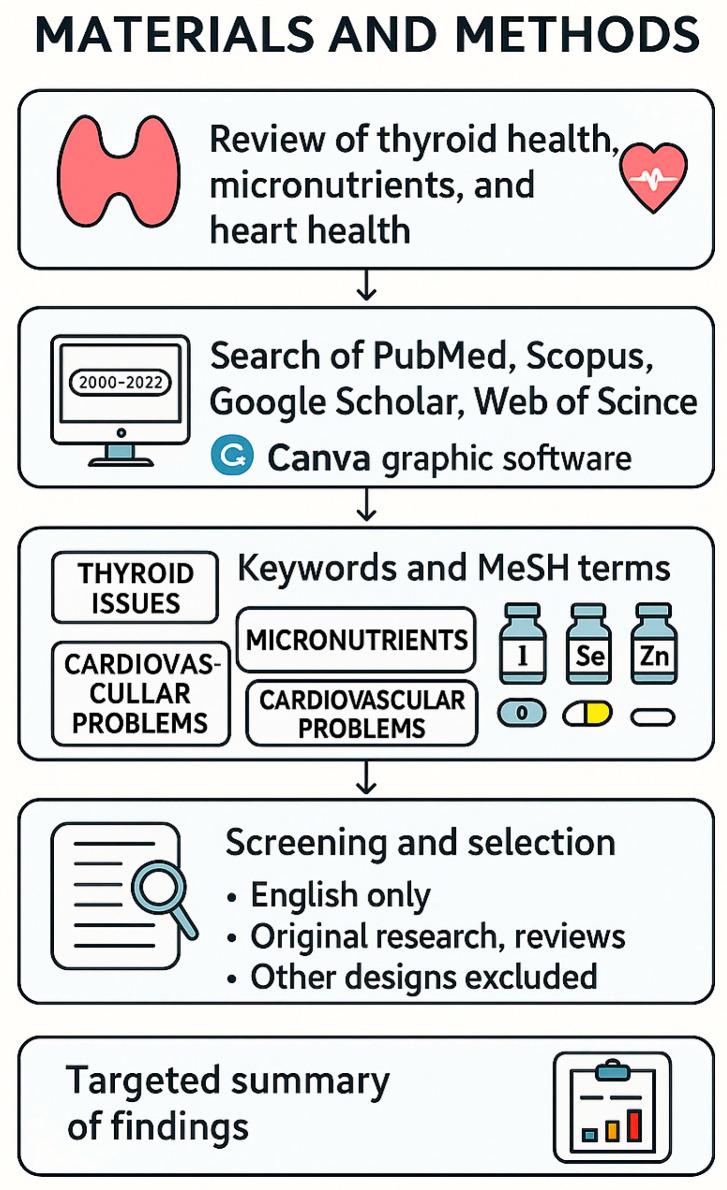
Flowchart outlining the methodology of the literature review on thyroid hormones, micronutrients, and cardiovascular health. The review process included source selection from major scientific databases, keyword identification, inclusion/exclusion criteria, and summarizing key findings. Illustration created using Canva graphic software.

To explore the relationship between thyroid function, micronutrient status, and cardiovascular health, a comprehensive literature review was conducted. The methodology included database searches, keyword selection, and eligibility screening. The literature search strategy is presented in Figure 2.

## 3. Sublinical Thyroid Dysfunction

Subclinical hyperthyroidism (SHyper) is characterized by suppressed TSH levels (<0.3–0.5 mIU/L), while subclinical hypothyroidism occurs with elevated TSH levels (>4.0–5.0 mIU/L), with both maintaining normal free T3 and T4 concentrations. Subclinical hypothyroidism is more common (3–10%) than SHyper (0.7–2%) [21,22]. The pathogenesis of subclinical thyroid dysfunction is often linked to autoimmune thyroiditis, particularly Hashimoto’s disease in the case of subclinical hypothyroidism [23]. At the molecular level, this involves lymphocytic infiltration of the thyroid gland, especially CD4+ and CD8+ T cells, leading to the progressive damage of thyrocytes. Proinflammatory cytokines such as interferon-γ (IFN-γ), interleukin-6 (IL-6), and tumor necrosis factor-alpha (TNF-α) play a key role in this immune-mediated destruction [24]. Oxidative stress also contributes to thyrocyte apoptosis, with reduced antioxidant defenses (e.g., glutathione peroxidase and superoxide dismutase activity) often observed in patients with autoimmune thyroiditis. Although hormone levels remain normal in the subclinical stage, elevated TSH levels reflect early pituitary compensation in response to declining thyroid reserve [25]. Subclinical hyperthyroidism, on the other hand, is often caused by autonomous thyroid hormone production, as seen in multinodular goiter or early Graves’ disease. In such cases, TSH receptor signaling becomes dysregulated, often due to TSH receptor-stimulating antibodies or somatic mutations in thyroid follicular cells, leading to increased thyroid hormone output despite suppressed TSH levels [26]. A study by Sun et al. [22] investigated the association between subclinical thyroid dysfunction and the risk of CVD. A meta-analysis of 16 studies showed that subclinical hypothyroidism generally did not increase the risk of most cardiac problems apart from mortality from ischaemic heart disease. This risk was more pronounced in those under 65 years of age. In contrast, SHyper was associated with an increased risk of ischaemic heart disease, mortality from it, and overall mortality [22].

The use of a diet represents a non-aggressive way that could bring concrete benefits. Studies highlighted the crucial importance of various nutrients such as anti-inflammatory nutrients—for example, vitamin D, antioxidants, mono- and polyunsaturated fatty acids, magnesium, and zinc—to reduce thyroid inflammation [27]. A lack of these nutrients could lead to a variety of health problems, such as anemia and CVD, especially in patients suffering from Hashimoto’s thyroiditis [28]. According to a study by Ruggeri et al. [29] which analyzed 200 individuals (81 with euthyroid Hashimoto’s thyroiditis and 119 healthy controls), patients with Hashimoto’s disease were found to consume significantly more animal products, particularly red and processed meat, compared to controls. A multivariable logistic regression model showed that a higher frequency of red and processed meat consumption was significantly associated with an increased risk of thyroid autoimmunity, as indicated by TPOAb positivity. In contrast, adherence to the Mediterranean diet was identified as a protective factor. The study also found elevated levels of oxidative stress markers, such as AGEs, and reduced activity of antioxidant enzymes, including GPx, GR, and TRxR, in Hashimoto’s patients. This suggests that a high intake of saturated fats and animal protein may contribute to redox imbalance and the development of autoimmunity. These findings support the hypothesis that limiting the intake of red and processed meat could help reduce oxidative stress and lower the risk of autoimmune thyroid disease [29].

Recent studies have emphasized the dysregulation of the Treg/Th17 axis in Hashimoto’s thyroiditis, with a shift toward proinflammatory Th17 responses (e.g., IL-17, IL-22) and reduced regulatory T cell activity. Selenium and vitamin D have been shown to restore Treg function and suppress Th17-driven autoimmunity, suggesting potential molecular pathways through which nutritional interventions could modulate disease progression.

## 4. Macronutrients in Thyroid and Cardiovascular Health

To improve clarity and avoid redundancy, this section consolidates the discussion of macronutrients and micronutrients involved in thyroid and cardiovascular regulation.

### 4.1. Protein

Animal and plant-based whole foods provide key nutrients relevant to thyroid and cardiovascular function. This may support weight regulation in some individuals, although interventional data are limited, and confounding factors—such as age, medication, or baseline metabolic state—must be considered [30]. In such cases, TSH levels might have been elevated due to the body’s adaptive response to a lack of protein and energy [31]. An increase in protein supply to 15–25% of the recommended total dietary energy could benefit health [32].

### 4.2. Carbohydrates

Whole-grain carbohydrates should be present in the diet, as they have a lower glycemic index, as well as more dietary fiber, vitamins, and minerals, compared to their wheat counterparts. A low glycemic index is also important for people with hypothyroidism, who may have disorders in carbohydrate metabolism. The presence of fiber in whole grain products can prevent constipation problems, which often occur in people with hypothyroidism. Fiber-rich whole grains aid metabolic control and support digestion in hypothyroid patients [33]. On the other hand, products rich in simple sugars, such as candies, cookies, waffles, high-sugar jams, and candied fruits, should be limited, as they increase the risk of obesity, diabetes, cancer, and other diseases [34].

### 4.3. Fats

People suffering from hypothyroidism and autoimmune thyroiditis should pay attention not only to the quantity, but also to the quality of fat consumed [30]. The World Health Organization (WHO) recommends that adults limit their total fat intake to 30% or less of their total energy intake. Such a recommendation aims to promote healthy lifestyles and reduce the risk of diseases associated with excessive fat consumption, such as obesity, cvd, and type 2 diabetes. It is crucial to pay attention to the fat present in dairy products and choose low-fat versions. Lipid profile alterations in hypothyroidism increase cardiovascular risk; the quality of fat intake is therefore important [35]. According to studies by Tzotzas T. et al. [36] and Teixeira et al. [37], changes in thyroid hormone levels in hypothyroidism affect total and LDL cholesterol levels. The results suggest that despite these changes, some other lipid parameters, such as triglycerides, apolipoprotein B, apolipoprotein A1, Lipoprotein (a), and quality abnormalities, may remain at similar levels or normalize. This is likely due to a more complex cause of dyslipidemia in hypothyroidism [36,37]. In contrast, a study by Sangeet et al. [38] analyzed lipid levels in patients with thyroid dysfunction and analysis of the association between these disorders and lipid profile. In the cross-sectional study, blood samples were collected from 112 patients with thyroid dysfunction and 100 healthy individuals. The results showed that patients with hypothyroidism had elevated levels of total cholesterol, LDL and triglycerides, and reduced levels of high-density lipoprotein (HDL), while patients with hyperthyroidism had lower levels of total cholesterol and triglycerides and higher levels of HDL compared to the control group. This may suggest that thyroid abnormalities may have a negative impact on patients’ lipid profile, which may increase the risk of CVD [38].

## 5. Relationship Between Nutrition and Hypothyroidism

**Figure 3:**
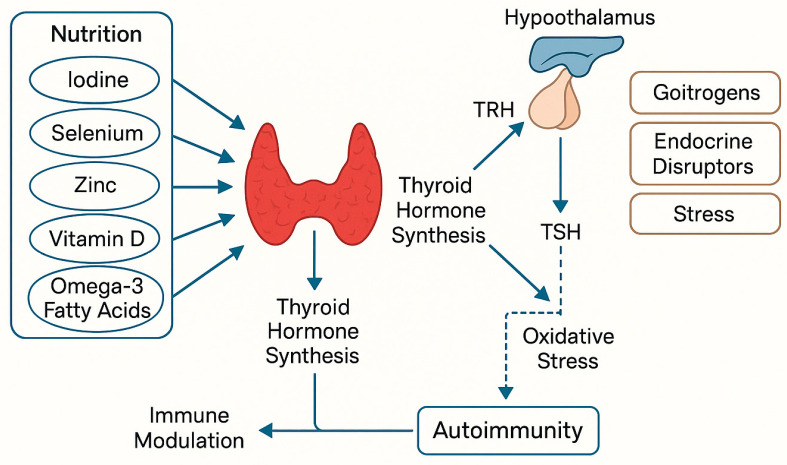
Nutritional and environmental factors influencing thyroid function and autoimmunity. Solid arrows indicate direct stimulation or contribution to thyroid hormone synthesis or immune modulation, while dashed arrows represent indirect effects such as stress-induced oxidative stress contributing to autoimmunity. (Original graphic created by the authors).

Proper nutrition plays a fundamental role in the management of hypothyroidism, particularly in patients with Hashimoto’s thyroiditis. This section discusses the impact of dietary habits and environmental exposures on metabolic health, weight regulation, and thyroid hormone activity, with attention to their combined influence on CVD risk [39]. A schematic representation of the molecular mechanisms through which nutrition affects thyroid function is shown in Figure 3, illustrating the interactions between nutrients, the immune system, and the hypothalamic–pituitary–thyroid (HPT) axis.

### 5.1. Metabolic Changes in Hypothyroidism

Hypothyroidism is associated with a decline in resting metabolic rate, which can result in decreased energy expenditure and progressive weight gain. Patients often report fatigue, cold intolerance, and reduced physical activity, further compounding weight-related challenges. The resulting metabolic dysregulation includes impaired thermogenesis, altered glucose and lipid metabolism, and a tendency toward insulin resistance—factors that also contribute to MetS and increased CVD risk [40,41]. Thyroid hormones influence key metabolic pathways, regulating the synthesis and breakdown of glucose, cholesterol, and triglycerides. They also play crucial roles in the cardiovascular system, nervous system, skeletal growth, and reproductive function, underscoring the systemic effects of hormonal imbalance [42].

### 5.2. Obesity and Body Composition

Weight gain is a common concern in hypothyroid patients, especially in Hashimoto’s disease, where autoimmune activity may coexist with metabolic dysfunction. An observational study by Valea et al. [43] found that 32% of 100 women with Hashimoto’s thyroiditis were classified as obese [43]. Similarly, Szwajkosz et al. [44] assessed the prevalence of excess weight in 101 patients with hypothyroidism, reporting that 39.6% were overweight, 26.7% had grade I obesity, and 10.9% had grade II obesity, with a mean body mass index (BMI) of 28.9. The study also found a correlation between age and increased risk of obesity, suggesting that older individuals with thyroid dysfunction may benefit most from early intervention and lifestyle counseling [44]. Given these findings, dietary management in hypothyroidism should be individualized. The goal is not aggressive caloric restriction, which can further suppress metabolism, but rather promoting sustainable weight control through balanced energy intake and increased physical activity, particularly in patients with coexisting obesity [40].

### 5.3. Environmental and Dietary Risk Factors

In addition to metabolic alterations, several environmental and dietary factors can negatively influence thyroid hormone production and increase the risk of hypothyroidism [45,46]. These include

- Iodine deficiency;
- Exposure to endocrine-disrupting chemicals (e.g., plastics, pesticides);
- Radiation exposure;
- Chronic psychological stress affecting the hypothalamic–pituitary–thyroid axis;
- Consumption of goitrogenic foods (e.g., raw cabbage, broccoli, cauliflower);
- Viral infections and pollutants contributing to autoimmune activation [47].

Understanding these factors is essential in identifying modifiable risks and shaping preventive strategies, especially for individuals with genetic predispositions to thyroid dysfunction [47].

### 5.4. Dietary Patterns Supporting Thyroid Health

While no single diet cures hypothyroidism, certain dietary patterns support thyroid function and reduce inflammation. Diets such as the Mediterranean, DASH, vegetarian, vegan, and hunter-gatherer diets emphasize the intake of nutrient-dense foods—especially fruits, vegetables, legumes, whole grains, nuts, and seeds—while limiting processed foods, added sugars, and sodium [48,49].

Among these, the Mediterranean diet is particularly promising due to its high content of selenium, omega-3 fatty acids, iodine, and antioxidants, all of which may help modulate immune responses and oxidative stress implicated in Hashimoto’s disease [50]. Furthermore, the 2015–2020 Dietary Guidelines for Americans encourage the consumption of a colorful variety of plant-based foods to promote health and reduce the risk of chronic diseases, which aligns well with dietary recommendations for thyroid patients [51].

## 6. Micronutrients in Thyroid and Cardiovascular Health

Micronutrient deficiencies, involving essential vitamins and minerals, pose a significant global health issue. According to the WHO, over 2 billion people worldwide suffer from deficiencies in micronutrients, which can lead to severe health consequences [52]. Simultaneously, micronutrient deficiencies often co-occur with overweight and obesity, further complicating disease profiles. Patients with heart failure (HF) frequently suffer from poor nutritional status due to factors such as decreased appetite, dietary restrictions, fatigue, dyspnea, nausea, or intestinal edema, which impair nutrient absorption and intake. These patients commonly lack key vitamins (A, B1, B2, and D) and minerals (calcium, magnesium, potassium, zinc, selenium, iodine, and copper), underscoring the need for systematic nutritional monitoring and intervention [53,54]. A deficiency in iodine, for example, can trigger autoimmune hypothyroidism by impairing the production of thyroid hormones (T3, T4), leading to goiter and the formation of antithyroid antibodies [53].

### 6.1. Iodine

Iodine is predominantly sourced from iodized salt, seafood (fish, seaweed), and selected cereals or baked goods [55]. During the 20th century, global efforts introduced universal salt iodization as a public health strategy against iodine deficiency disorders—a measure that proved highly effective. However, with rising concerns over hypertension and CVD, recommendations to reduce sodium intake have created a public health dilemma: how to maintain sufficient iodine intake while limiting salt consumption. The WHO recommends 150 µg of iodine per day for adults [56]. The thyroid gland stores 15–20 mg of iodine—around 70–80% of the body’s total iodine pool—while approximately 80 µg is utilized daily for hormone synthesis. Intake below 50 µg can cause deficiency, whereas chronic intake above 350 µg (especially > 600 µg) may impair thyroid function [57]. Maintaining iodine homeostasis within this narrow therapeutic window is thus crucial. In Poland, where the average salt consumption exceeds 13.5 g/day (far above the WHO-recommended <5 g/day), excessive sodium intake contributes to increased CVD risk. The Polish Society of Hypertension recommends salt restriction, especially in high-risk groups such as the elderly, diabetics, and patients with MetS or chronic kidney disease [58]. Studies like that by Kypridemos et al. [59] demonstrate that sodium reduction policies can significantly reduce CVD incidence and mortality [59]. Nonetheless, iodized salt remains a major dietary iodine source. Therefore, striking a balance between iodine sufficiency and sodium limitation is vital. Alternative iodine sources—such as fortified dairy products, sea vegetables, or potassium iodate supplementation in other foods—may help address this issue [60]. For instance, the Korean cohort study by Park et al. [61], involving over 190,000 adults, found no direct correlation between high iodine intake and thyroid disease after adjusting for confounding factors, suggesting population-specific tolerability levels and the need for further research [61]. Supplementary Table S1 [62,63] shows the iodine levels in selected foods, highlighting the need for a balanced intake of iodized salt and other sources of iodine in the daily diet in the context of health prevention and CVD prevention.

### 6.2. Vitamin D

Vitamin D is synthesized endogenously through skin exposure to sunlight and can also be obtained from diet and fortified products. In Poland, vitamin D-fortified foods (e.g., cereals, dairy drinks, and juices) are widely available, and their inclusion may increase intake up to 3.5-fold. It is important, however, to select products with low sugar, saturated fat, and sodium content [64,65].

Dietary vitamin D is richest in fatty fish (e.g., European eel, herring), while smaller amounts are found in eggs and offal. Vitamin D deficiency is common in individuals with autoimmune thyroid diseases, possibly due to genetic or environmental factors. Importantly, vitamin D contributes to cardiovascular health by modulating blood pressure and stabilizing atherosclerotic plaques. Supplementary Table S2 [66] lists selected vitamin D-rich food sources.

### 6.3. Iron

Iron deficiency is particularly prevalent among individuals with Hashimoto’s thyroiditis due to impaired nutrient absorption and increased needs associated with chronic inflammation [28]. Iron supports hemoglobin production and is essential for thyroid hormone synthesis, primarily through its role in heme-dependent enzymes [67]. Rich dietary sources include

- Red meat and liver—highly bioavailable heme iron;
- Cocoa and dark chocolate—non-heme iron, enhanced by vitamin C;
- Spinach—iron-rich, though less bioavailable;
- Sardines and seafood—provide iron along with omega-3s;
- Pumpkin seeds—a plant-based source [40].

In Denmark, mandatory iodization of table salt and salt used in bread baking was introduced to prevent iodine deficiency, which can cause various health issues, including thyroid disorders. However, following the implementation of this regulation, an increase in autoimmune thyroid diseases has been reported [68]. A study conducted between 1992 and 2005 involving over 1400 children found that iodized salt helped reduce the prevalence of goiter and increased urinary iodine concentrations in this population. While the authors referenced the Danish findings, they also highlighted a rising incidence of thyroiditis [69]. Another study emphasized the need for further research to clarify the role of diet in Hashimoto’s disease [52]. Studies indicate that diets rich in heme iron are associated with a higher risk of CVD and related mortality compared to diets relying on plant-based iron sources. These differences may be explained by the presence of phytochemicals and the overall food matrix. Plant-based foods lacking heme iron are typically rich in antioxidants, which benefit heart health. In contrast, heme iron sources such as meat often contain long-chain saturated fatty acids, which are linked to CVD. Moreover, emerging research suggests that metabolites of heme iron may increase CVD risk by promoting inflammation and adversely altering blood lipid profiles. Collectively, these findings imply that the source of dietary iron plays a significant role in cardiovascular health [70]. Interestingly, Cleland et al. [71] reported that elevated ferritin levels—not low levels—are associated with increased overall mortality and cardiovascular deaths, challenging the conventional link between ferritin concentration and iron availability in heart failure patients. Furthermore, a subgroup analysis from a meta-analysis of 839 patients, drawn from four double-blind, randomized controlled trials, demonstrated that intravenous administration of ferric carboxymaltose (FCM) significantly reduced cardiovascular hospitalizations and mortality in this population. Notably, patients with transferrin saturation (TSAT) levels below 20.1% benefited more from FCM therapy than those with TSAT levels above this threshold, even when ferritin levels were low [71]. Animal models and transplant studies have shown that myocardial iron deficiency impairs cardiac contractility and worsens outcomes in advanced heart failure [72,73].

### 6.4. Selenium

Selenium is an essential trace element crucial for numerous biological functions. It supports antioxidant synthesis, boosts immune defense, and primarily benefits thyroid health, cardiovascular regulation, neurodegenerative disease prevention, and infection resistance [74,75]. Dietary intake remains the main source of selenium, which has prompted considerable attention toward enriching foods with this nutrient. Reflecting this, the National Institutes of Health Office of Dietary Supplements lists over 30 selenium-containing food sources, demonstrating progress and the variety available to consumers [76]. Recommended dietary intake (RDI) varies worldwide; for example, adult women in Japan require 25 μg/day, while in the Netherlands and Macedonia, recommendations approach or exceed 100 μg/day. These disparities likely stem from regional dietary habits, food availability, and population health needs [60]. Generally, the Recommended Dietary Allowance for adults ranges between 55 and 70 μg/day. Deficiency is more common in parts of Europe and China due to selenium-poor soils limiting its presence in local foods [54,77,78].

Selenium concentrations in typical foods are listed in Supplementary Table S3 [79,80], with Brazil nuts (160 μg/100 g), oysters (63.6 μg/100 g), and canned tuna (60.1 μg/100 g) being particularly rich sources. Moderate amounts are found in pasta and pork, while tofu, octopus, white rice, garlic, and pumpkin contain lower levels.

Selenium is integral to selenoprotein formation, which regulates redox balance, cell proliferation, apoptosis, thyroid hormone metabolism, and cardiovascular function [81]. Deficiency can cause disorders such as Keshan disease, a cardiomyopathy seen in selenium-deficient Chinese regions [82]. Favaro et al. [83] found that regional intake differences were noted in Brazil, where daily selenium ranged from 20 to 114 μg—influenced by socioeconomic status and diet—with cereals, fish, and meat as primary sources [83]. Rasmussen et al. [84] found that low serum selenium levels correlated with enlarged thyroid volume and increased nodules in moderately iodine-deficient Danish areas, both before and after iodine fortification [84]. In a study by Socha et al. [85], 137 Hashimoto’s patients showed significantly lower mean serum selenium (63.03 µg/L) levels compared to controls (75.16 µg/L, *p* < 0.0007). Frequent intake of eggs, meat, and legumes was linked to higher selenium concentrations, while margarine and fish consumption inversely affected selenium levels, although their overall dietary influence was modest. In conclusion, maintaining balanced selenium intake is critical for antioxidant defenses, thyroid hormone regulation, and immune function, especially in autoimmune thyroid disease. Both deficiency and excess present health risks, underscoring the need for well-informed nutritional strategies [85].

### 6.5. Copper

Copper is an indispensable trace mineral involved in many physiological processes and is abundant in foods such as meat, fish, shellfish, seeds, whole grains, chocolate, and leafy vegetables. The RDI is approximately 1 mg for both adult men and women [86]. Copper acts as a vital cofactor for tyrosinase and plays a key role in thyroid hormone metabolism, including the conversion of inactive T4 to active T3. This process is essential for synthesizing thyroperoxidase (TPO), the enzyme responsible for thyroid hormone production and iodothyrosine coupling, which regulates metabolism and physiological functions [87]. A cohort study by Blasig et al. [88] involving 84 children with congenital hypothyroidism (CH) demonstrated the importance of sufficient copper levels for thyroid function and growth. The average serum copper concentration was within normal ranges (1384.2 ± 388.8 μg/L), and a strong positive correlation was observed between copper and thyroid hormones. Though no association with selenium was noted, copper deficiency might impair development in severe hypothyroidism, suggesting copper could be a biomarker for monitoring thyroid hormone replacement therapy efficacy in pediatric patients; larger studies are warranted [88]. In a clinical investigation by Maouche et al. [89] of 220 adults with thyroid disorders and 50 healthy controls, correlations emerged between thyroid dysfunction, insulin resistance, obesity, dyslipidemia, and oxidative stress, highlighting trace element imbalances, particularly copper’s role in disease pathophysiology [89]. Similarly, Stojsavljević et al. [90] compared 23 hypothyroid patients with 70 controls, finding significantly elevated copper levels (*p* < 0.0001) in the patient group, indicating that altered copper status may serve as a diagnostic marker for thyroid disorders [90].

Supplementary Table S4 [91,92] details copper-rich foods, with cocoa powder providing the highest amount (3.71 mg/100 g), followed by sunflower seeds (1.87 mg) and pumpkin seeds (1.57 mg). Other moderate sources include hazelnuts, almonds, and fresh parsley. Ten grams of cocoa powder alone supplies 0.37 mg of copper, making it an excellent dietary source, while cereal products and whole-grain bread contain lower amounts.

In summary, copper is essential for thyroid hormone metabolism, oxidative balance, and endocrine health. Its serum levels are influenced by dietary intake and thyroid disease status, supporting its potential use as a clinical biomarker.

### 6.6. Zinc

Zinc deficiency can reduce circulating levels of thyroid hormones T3 and T4, increasing the risk of hypothyroidism. Therefore, sufficient zinc intake is critical for proper hormonal balance and thyroid function [93]. Hair loss is a common symptom linked to zinc deficiency in hypothyroid individuals, as zinc is vital for hair growth and regeneration [94]. Foods rich in zinc include pumpkin seeds, flax seeds, wholemeal cereals like wholemeal bread, millet, and buckwheat groats. In Poland, cereals contribute the most to dietary zinc, while meat products supply smaller amounts [95]. The recommended daily allowance (RDA) is 8 mg for women and 11 mg for men [96]. Zinc is important in maintaining thyroid homeostasis, influencing hormone synthesis and activation at multiple levels. It regulates deiodinase enzymes responsible for thyroid hormone conversion, affects both the secretion and production of TSH, and participates in hormone synthesis via the modulation of transcription factors [85]. Patients with idiopathic dilated cardiomyopathy exhibit elevated copper but reduced serum zinc compared to healthy controls, supporting previous findings linking zinc deficiency to cardiac health [82]. Zinc deficiency disrupts heart cell structure and function, positioning zinc levels as potential diagnostic and prognostic markers in chronic cardiomyopathies [97]. While earlier beliefs suggested zinc intake might protect against hypertension, recent cohort studies indicate that higher zinc consumption is associated with a lower risk of hypertension but simultaneously linked to increased diabetes risk [98]. The connection between serum zinc and CVD has been explored in several studies. For example, Soinio et al. [99] found that type 2 diabetes patients with low serum zinc levels had a higher incidence of cardiovascular events, emphasizing the importance of zinc monitoring [99]. Conversely, Joo et al. [99,100] reported increased mortality in CVD patients with low zinc, suggesting zinc supplementation may benefit those with chronic metabolic disorders [100].

Supplementary Table S5 [64] lists zinc-rich foods important for thyroid hormone production and cardiovascular well-being. Calf liver contains the highest zinc amount (84 mg per 1000 g), followed by pumpkin seeds (0.75 mg per 10 g), dark cocoa powder (0.66 mg per 10 g), and pork liver (4.51 mg per 100 g). Dried white beans and buckwheat groats provide moderate zinc, while nuts and cereals such as almonds, oatmeal, and whole-grain rye bread contribute moderate levels. Eggs, white rice, fresh cod, and tomatoes contain smaller quantities. A summary of these studies is available in Supplementary Table S6 [9,10,11,56,59,71,83,84,85,88,89,90,99,100].

**Figure 4:**
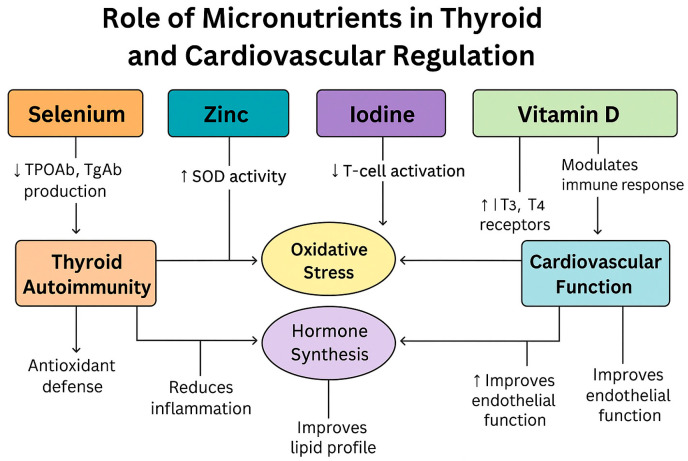
Role of micronutrients in the regulation of thyroid autoimmunity, oxidative stress, hormone synthesis, and cardiovascular function. The arrows indicate the direction of influence or effect between micronutrients and physiological processes (e.g., Selenium reduces TPOAb/TgAb production, thereby influencing thyroid autoimmunity). Original graphic created by the authors using elements and icons licensed under Canva subscription. No third-party copyrighted content was used.

The combined molecular effects of selenium, iodine, vitamin D, and zinc on thyroid and cardiovascular function are summarized in Figure 4.

## 7. Conclusions

Thyroid dysfunction, particularly hypothyroidism and Hashimoto’s thyroiditis, is closely linked to increased cardiovascular risk through mechanisms involving immune dysregulation, oxidative stress, and hormonal imbalance. This review highlights the critical role of micronutrients—especially selenium, iodine, zinc, vitamin D, and iron—in supporting thyroid function and modulating these pathogenic pathways. We propose a novel integrative framework that links nutritional status with immune pathways (e.g., Treg/Th17 imbalance), hormone synthesis, and cardiovascular outcomes, emphasizing sex-specific and personalized dietary strategies. The involvement of non-genomic mechanisms—especially MAPK and PI3 K/Akt signaling—in mediating the vascular and mitochondrial effects of T3 further underscores the importance of trace elements that regulate these pathways.

Although the reviewed associations are promising, most evidence is observational. Future research should focus on randomized controlled trials stratified by sex, nutritional status, and comorbidities. Understanding how nutrient–immune–hormone interactions contribute to cardiovascular outcomes may lead to novel, personalized prevention strategies in endocrine and cardiometabolic care.

## Data Availability

No new data were created or analyzed in this study. Data sharing is not applicable to this article.

## Supplementary Materials

The following supporting information can be downloaded at https://www.mdpi.com/article/10.3390/pathophysiology32030037/s1. Table S1: Iodine in selected foods; Table S2: Vitamin D content in selected foods; Table S3: Animal and vegetable products with high selenium content per 100 g of product; Table S4: Examples of products with copper content per 100 g of product; Table S5: Examples of products with zinc content per 100 g of product, Table S6: Studies focused on micronutrients in thyroid diseases and CVD.

## Abbreviations

The following abbreviations are used in this manuscript:

OH	Overt Hypothyroidism
CVD	Cardiovascular Disease
TSH	Thyroid-Stimulating Hormone
T4	Thyroxine
T3	Triiodothyronine
BMI	Body Mass Index
HDL	High-Density Lipoprotein
LDL	Low-Density Lipoprotein
WHO	World Health Organization
TSAT	Transferrin Saturation
FCM	Ferritin Carboxymaltose
MetS	Metabolic Syndrome
TPO	Thyroid Peroxidase
TPOAbs	Thyroid Peroxidase Antibodies
RDI	Recommended Daily Intake
CH	Congenital Hypothyroidism
TgAbs	Thyroglobulin Antibodies
SHyper	Subclinical hyperthyroidism
FCM	Ferric Carboxymaltose
HPT Axis	Hypothalamic–Pituitary–Thyroid Axis
ROS	Reactive Oxygen Species
MAPK	Mitogen-Activated Protein Kinase
PI3K/Akt	Phosphoinositide 3-Kinase/Protein Kinase B Pathway
NO	Nitric Oxide
