# Supplementary material for: Nutrition and Micronutrient Interactions in Autoimmune Thyroid Disorders: Implications for Cardiovascular Health
Source: Pathophysiology. 2025 Aug 1;32(3):37. doi: 10.3390/pathophysiology32030037 (PMC12372124; doi:10.3390/pathophysiology32030037)
Supplement: Supplementary file 1 [file pathophysiology-32-00037-s001.zip › pathophysiology-3743790-supplementary.pdf]

Table S1. Iodine in selected foods [62,63].

Product	Iodine Content 100 g (μg)	Product Portion	Iodine Content per Serving (μg)
Common salt, iodised	2293,0	1 teaspoon (6 g)	137,58
Fresh cod	110,0	1 piece (100 g)	110
Fresh pollock	103,0	1 piece (100 g)	103
Smoked mackerel	40,0	1 piece (350 g)	140
Wheat bran	31,0	1 spoon (4 g)	1,24
Sardine in tomatoes	25,0	1 serving (120 g)	30
Tuna in oil	17,0	1 piece (170 g)	28,9
Hazelnuts	15,0	1 handful (30 g)	4,5
Broccoli	12,0	1 flush (20 g)	2,4
Spinach	9,5	1 handful (25 g)	2,375
Kefir 2% fat	7,5	1 package (240 g)	18
Whole hen eggs	5,9	1 piece (50 g)	2,95
Oatmeal	5,7	1 spoon (10 g)	0,57
Rye flakes	5,7	1 spoon (10 g)	0,57
Banana	2,8	1 piece without peel (120 g)	3,36
Wholemeal rye bread	2,7	1 slice (35 g)	0,945
White rice	2,2	1 spoon (15 g)	0,33
Apple	1,6	1 piece (200 g)	3,2
Tomato	1,1	1 piece (170 g)	1,87

Table S2. Vitamin D content in selected foods [66].

Product	Vitamin D Content 100 g (μg)	Product Portion	Vitamin D Content per Serving (μg)
Smoked eel	36,00	1 piece (40 g)	14,4
European ell	30,00	1 piece (3600 g)	1080
Herring in oil	20,22	1 piece (70 g)	14,154
Smoked herring	20,00	1 piece (135 g)	27
Herring	19,00	1 piece (135 g)	25,65
Herring in tomato sauce	15,48	1 piece (170 g)	26,316
Rainbow trout	13,60	1 piece (250 g)	34
Salmon	13,00	1 piece (200 g)	26
Smoked salmon	13,00	1 piece (100 g)	13
Smoked mackerel	8,40	1 piece (250 g)	21
Tuna in oil	5,80	1 piece (170 g)	9,86
Sardines in oil	5,00	1 piece (90 g)	4,5
Yolk of a hen's egg	4,5	1 piece (15,5 g)	0,6975
Hen’s egg	1,70	1 piece (55 g)	0,935
Pork liver	1,10	1 piece (100 g)	1,1
Extra butter	0,76	1 teaspoon (10 g)	0,076
Pork shoulder, pork neck	0,70	1 piece (200 g)	1,4
Turkey breast meat with skin	0,30	1 carcass (1700 g)	5,1

Table S3. Animal and vegetable products with high selenium content per 100 g of product [79,80].

Product	Selenium Content 100 g (µg)	Product Portion	Selenium Content per Serving(µg)
Brazil nuts	160	1 handful (50 g)	80
Oysters	63,6	100 g	63,6
Tuna in oil (after draining)	60,1	1 tin (185 g)	111,18
Pasta	57,5	1 tsp (15 g)	8,63
Pork, leg	54,8	100 g	54,8
Clams	44,8	1 piece (100 g)	44,8
Octopus	44,8	fresh 1 piece (50 g)	22,4
Parmesan	38,65	1 tsp (9 g)	3,48
Wheat flour	35,3	1 tsp (12 g)	4,24
Oatmeal	28,3	1 tsp (10 g)	2,83
Turkey leg	23	1 piece (160 g)	36,8
Raw cod	19	1 piece (100 g)	19
Tofu	16,7	1 tsp (20 g)	3,34
Mozzarella light	16,1	1 piece (125 g)	20,13
White rice	15,1	1 tsp (15 g)	2,27
Garlic	14,3	1 tsp (8 g)	1,14
Pumpkin seeds, roasted	9,32	1 tsp (8 g)	0,74
Broccoli	4,9	1 flush (20 g)	0,98

Table S4. Examples of products with copper content per 100 g of product [91,92].

Product	Copper Content 100 g (mg)	Product Portion	Copper Content per Serving (mg)
Cocoa 16%, powder	3,71	1 teaspoons (10 g)	0,37
Sunflower seeds	1,87	1 spoon (10 g)	0,19
Pumpkin seeds	1,57	1 spoon (10 g)	0,16
Hazelnuts	1,29	1 spoon (15 g)	0,19
Almonds	1,00	1 spoon (15 g)	0,15
Parsley	0,61	1 spoon (6 g)	0,04
Oatmeal	0,55	1 spoon (10 g)	0,06
Buckwheat groats	0,41	1 spoon (10 g)	0,04
Walunts	0,30	1 spoon (10 g)	0,03
Wholemeal rye bread	0,24	1 slice (40 g)	0,10
Graham rolls	0,22	1 piece (65 g)	0,14

Table S5. Examples of products with zinc content per 100 g of product [64].

Product	Zinc Content 100 g (mg)	Product Portion	Zinc Content per Serving (mg)
Calves' liver	8,40	1 serving (1000 g)	84,00
Pumpkin seeds	7,50	1 spoon (10 g)	0,75
Cocoa 16%, powder	6,56	1 teaspoons (10 g)	0,66
Pork liver	4,51	1 piece (100 g)	4,51
White beans, dry seed	3,77	1 handful (40 g)	1,51
Buckwheat groats	3,50	1 spoon (10 g)	0,35
Almonds	3,19	1 spoon (15 g)	0,48
Oat flakes	3,10	1 spoon (10 g)	0,31
Wholemeal rye bread	2,54	1 slice (35 g)	0,89
Hazelnuts	2,44	1 handful (30 g)	0,73
Bitter chocolate	2,43	1 cube (6 g)	0,15
Whole hen eggs	1,76	1 piece (50 g)	0,88
White rice	1,73	1 spoon (15 g)	0,26
Fresh cod	0,50	1 piece (100 g)	0,50
Tomato	0,26	1 piece (170 g)	0,44

Table S6. Studies focused on micronutrients in thyroid diseases and CVD.

Study	Participants	Key Findings	Focus
WHO Guidelines [56]	General population	Recommended daily iodine intake for adults is 150 μg/day.	Iodine intake and thyroid health
Kypridemos et al. [59]	UK population	Reducing NaCl intake (2003-2015) prevented/delayed CVD in 52,000 people and saved 10,000 lives.	Salt reduction and CVD prevention
Naliwajko et al. [9]	96 women with Hashimoto's disease (Poland)	Average iodine intake was close to EAR (0.095 mg/day), but >1/3 of participants had insufficient iodine intake.	Iodine intake in Hashimoto's patients
Cleland et al. [71]	839 patients with heart failure	Intravenous ferritin carboxymaltose (FCM) reduced hospitalizations and mortality in heart failure patients, especially those with TSAT <20.1%.	Iron supplementation in heart failure
Rasmussen et al. [84]	805 participants (Denmark)	Low selenium levels were associated with increased thyroid volume and risk of thyroid nodules.	Selenium and thyroid health
Socha et al. [85]	137 patients with Hashimoto's thyroiditis	Mean selenium concentration was 63.03 μg/L (vs. 75.16 μg/L in controls). Dietary habits and smoking influenced selenium levels.	Selenium levels in Hashimoto's patients
Blasig et al. [88]	84 children with congenital hypothyroidism	Mean serum copper concentration was 1384.2 ± 388.8 μg/L. Strong positive correlation between copper levels and thyroid hormones.	Copper and thyroid hormone levels in children
Maouche et al. [89]	220 adults with thyroid disorders (Algiers)	No significant differences in copper levels between hypothyroid and control groups.	Copper and oxidative stress in thyroid disorders
Stojsavljević et al. [90]	93 patients (23 with hypothyroidism, 70 healthy)	Copper levels were significantly higher in hypothyroid patients ($p < 0.0001$).	Copper as a biomarker for hypothyroidism0,89
Soinio et al. [99]	Patients with type 2 diabetes	Low serum zinc levels were associated with increased cardiovascular events.	Zinc and CVD risk in diabetes
Joo et al. [100]	CVD patients	Low serum zinc levels correlated with increased mortality. Zinc supplementation may benefit chronic metabolic disease patients.	Zinc and mortality in CVD[8]
Ghasemi et al. [10]	Men and women with metabolic syndrome (Tehran)	Higher zinc levels in men were associated with increased risk of MetS, while in women, higher zinc levels were associated with lower risk.	Zinc and metabolic syndrome
Vinceti et al. [11]	680 adults (Punjab)	High selenium exposure correlated with increased blood pressure and hypertension, especially in women.	Selenium and hypertension
Favaro et al. [83]	Brazilian population	Selenium intake ranged from 20 to 114 μg/day, depending on region and socioeconomic level.	Selenium intake in Brazil
